# Effects of Nursing Diabetes Self-Management Education on Glycemic Control and Self-Care in Type 1 Diabetes: Study Protocol

**DOI:** 10.3390/ijerph19095079

**Published:** 2022-04-21

**Authors:** Rocío Romero-Castillo, Manuel Pabón-Carrasco, Nerea Jiménez-Picón, José Antonio Ponce-Blandón

**Affiliations:** Red Cross Nursing School, Centro Universitario de Enfermería de Cruz Roja, Universidad de Sevilla, Avenida de la Cruz Roja, nº 1, 41009 Seville, Spain; rocio.romero@cruzroja.es (R.R.-C.); nejipi@cruzroja.es (N.J.-P.); japonce@cruzroja.es (J.A.P.-B.)

**Keywords:** type 1 diabetes, health education, diabetes education, nurses, advanced nursing, self-management, self-care, metabolic control, glycemic control

## Abstract

(1) Background: Type 1 diabetes is a chronic disease that creates a high demand and responsibility for patient self-care. Patient education, self-care training and the management of derived complications are great challenges for nurses. The objective of this project is to evaluate the efficacy of a therapeutic education program for type 1 diabetes. (2) Methods: Participants recruited to the study will be adult patients with diagnosed type 1 diabetes attending the clinic at the study site. A nurse diabetes educator will deliver a four-session education program. A two-group randomized controlled trial will be used in this study, with an intervention group and a control group. The subjects included in the experimental group will attend some health education sessions, while control group participants will receive the existing standard care provided by the endocrinology and nutrition unit of the hospital. Measurements and evaluations will be conducted at the baseline prior to the intervention and at 1 and 3 months from the intervention. (3) Conclusions: The primary outcome is improving patients’ knowledge about diet and treatment management. Secondary outcomes are improving patients’ glycemic control and mood. The findings from this study will help to determine the effect of diabetes education about self-care and treatment in patients with diabetes, as well as helping to decrease short-term and long-term complications and reduce health care costs.

## 1. Introduction

Type 1 diabetes is an autoimmune disease affecting 1 in every 300 persons, and the number of newly diagnosed cases is growing [1]. The number of people living with diabetes is expected to continue to increase, affecting an estimated 550 million by 2030. Diabetes is a prominent public health concern associated with increased premature mortality [2]. Long-term microvascular and macrovascular complications in patients with type 1 diabetes have been shown to be reduced with optimal glucose control [3]. In addition to pharmacological treatment, the importance of lifestyle modification, mainly diet, has been demonstrated [4]. Evidence has suggested that diabetes self-management education improves short-term glycemic control and reduces diabetes complications. Practice nurses are in a good position to provide monitoring, tailored feedback and health education [5]. 

The American Diabetes Association 2015 *Standards of Medical Care in Diabetes* recognizes diabetes self-management education (DSME) as a holistic aspect of the care of people with this chronic pathology [6,7]. Diabetes self-management education is defined as a continued facilitation of the development of the knowledge and abilities necessary for the optimal self-management of diabetes [8]. Evidence suggests that DSME is cost-effective [9] and associated with favorable changes in knowledge, clinical outcomes, self-efficacy and other psychosocial outcomes, screening for complications, risk factors for cardiovascular events and quality of life [10,11,12].

The recommended treatment regimen for type 1 diabetes is complex, and it requires the frequent monitoring of blood glucose levels, the control of carbohydrate intake, frequent insulin administration and participating in moderate-intensity physical activity [6]. Type 1 diabetes is a chronic disease that creates a high demand and responsibility for patient self-care. Patient education, self-care training and the management of derived complications are great challenges for nurses [13].

Four or more capillary blood glucose tests are required daily to safely and effectively adjust insulin doses. It is often difficult to perform the necessary daily check-ups due to pain from the finger-stick. Over time, patients develop calluses on their fingertips due to numerous punctures [14]. Owing to the progression of technology, in recent years sensors have been created that allow glucose monitoring, such as the FreeStyle Libre (FSLCGM Abbott Diabetes Care, Alameda, CA, USA). This rapid glucose monitoring system provides the mechanical reading and continuous measurement of interstitial fluid glucose concentration and provides the corresponding ambulatory glucose profile. Patients can scan their sensor with a reader or with their personal mobile device through an application. They can meet target glucose levels and average periods of hypoglycemia and hyperglycemia, as well as set alarms to notify them of high or low blood glucose levels [15,16].

This paper describes a study protocol for analyzing the influence of therapeutic education in the treatment management and self-care of patients with type I diabetes. To assess whether education influences glucose control, we will review the records of the continuous glucose monitoring sensor. We will compare the sensor parameters before and after the educational intervention, such as time in the optimal glycemic range, time in hypoglycemia or hyperglycemia and average glucose level. A pre-/post-intervention test of knowledge on the management of the disease will be administered to assess the knowledge acquired after the therapeutic education. Finally, we will analyze whether the change in the level of knowledge and in glycemic control influences or has any repercussions for the emotional state of the patient.

## 2. Materials and Methods

### 2.1. Design

There will be two groups in this randomized controlled trial project, an intervention group and a control group. Patients included in the intervention group will participate in four sessions of therapeutic education on the management of diabetes, while the subjects included in the control group will have access to the standard care provided by the endocrinology and nutrition unit of the hospital. The administration of tests and questionnaires as well as the evaluation of the glycemic record will be conducted at the baseline and at 1 and 3 months from the intervention. 

### 2.2. Population and Sample

This study is aimed at patients who came to the endocrinology and nutrition unit with a diagnosis of type 1 diabetes mellitus. Participants will be recruited during an endocrinology consultation with a medical professional specializing in diabetes. They will be recruited through random sampling at a public hospital in Spain. 

Randomization in blocks of 10 will be used (five experimental and five controls) to achieve balance in the randomization procedure. A person unrelated to the research objectives and data collection will prepare the sealed envelopes according to the generated randomization list. A unique patient ID will be assigned, and each patient will be allocated into one of the two groups. 

The sample size will be calculated based on Cohen’s (1992) recommendation for the expected differences between groups. Previous studies that examined the effect of diabetes self-management education on glycemic control and health-related self-care in patients with diabetes suggested a medium effect size. Given the medium effect size (0.5), a repeated-measures analysis of covariance will be performed to test for differences between the two groups, and approximately 176 participants will be required to achieve a 95% power at a 5% level of significance [17].

### 2.3. Eligibility Criteria

Patients must meet the following inclusion and exclusion criteria to be eligible for the study.

#### 2.3.1. Inclusion Criteria

- Adults over the age of 18 years.
- Diagnosed with type 1 diabetes.
- Able to speak, read and understand Spanish.

#### 2.3.2. Exclusion Criteria

- Have a terminal illness.
- Have at least one of the following clinical conditions or pathologies: brain injury of traumatic or hemorrhagic origin, dementia or serious mental illness such as schizophrenia.
- Have reading and hearing difficulties.

All participants will be required to complete written informed consent. The inclusion and exclusion criteria will be similar to those used in previous studies, following current scientific evidence in this population [4,18]. 

### 2.4. Intervention

#### 2.4.1. Diabetes Education Program

**Figure 1:**
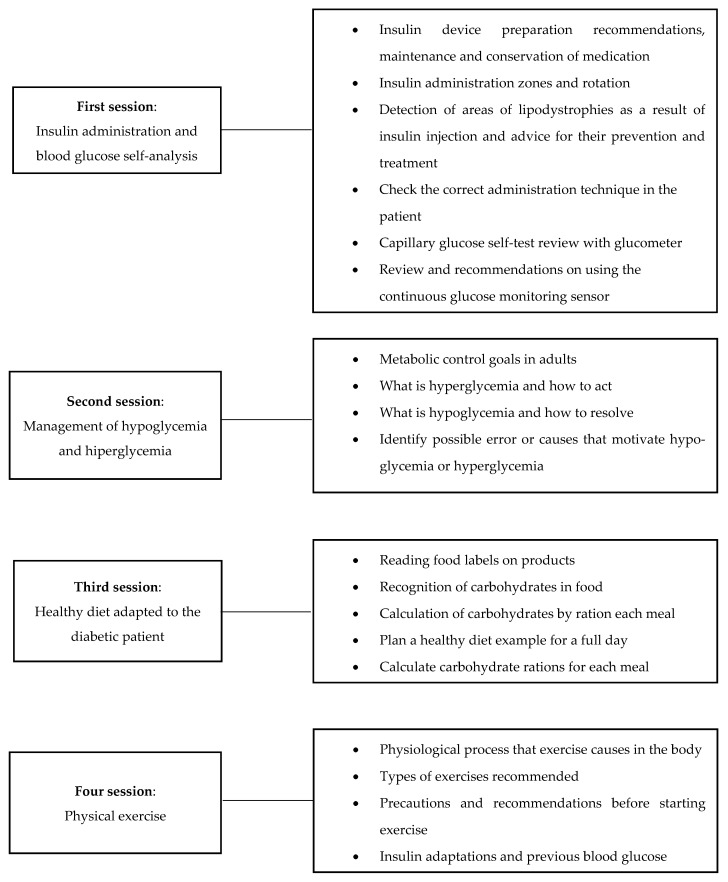
Structure of the diabetes education program.

Participants randomized to the intervention group will receive a structured program of therapeutic education organized over four consecutive days. The organization of the sessions is shown in Figure 1. 

The therapeutic education will be provided by an advanced practice nurse specializing in diabetes. The sessions will be one hour a day with clear and concise information to guarantee the attention and concentration of the participants. This education program offers advanced and centralized training in diabetes care.

#### 2.4.2. Usual Care

Participants in the control group will receive the usual services offered in the endocrinology and nutrition unit of the university hospital, which include regular visits with a doctor specializing in endocrinology and with a nurse educator and standard Spanish Diabetes Society information pamphlets. Consultation care will be centralized in the pharmacological treatment regimen, dosage and guidelines.

### 2.5. Data Collection

Data collection will occur at three different time points throughout the study: at the baseline, 1 month and 3 months. The baseline visit will be in person, and subsequent visits can be either in person or over the phone. Sociodemographic data, such as gender, age, education level and employment status, will be collected at the baseline. The following parameters and scales will be collected at all three points (baseline, 1 month and 3 month).

#### 2.5.1. Blood Glucose Levels According to Continuous Glucose Monitoring Sensor

Type 1 diabetes patients in the endocrinology and nutrition unit have the opportunity to benefit from the free, hospital-funded sensor for continuous glucose monitoring. The sensor is implanted in the back of the arm; it has a microfilament that allows measuring glucose in the interstitial fluid. Patients must change their sensor every 14 days. The glucose levels can be visualized by both a specific reader and a mobile application based on near-field communication (NFC) technology.

This is an objective report on the evolution in glycemic control after receiving training in the diabetes education program. The nurse will have access to these data, and the parameters to be evaluated will be the following: times in high range (>181 mg/dL), target range (70–180 mg/dL) or low range (<69 mg/dL); average glucose (mg/dL); and glucose management indicator (%).

#### 2.5.2. General Knowledge about Diabetes Management

A brief knowledge test was administered to each patient. The test consisted of ten short questions to test their basic knowledge about diet and insulin management. This questionnaire will help us to know if the patient’s knowledge improves after participating in the therapeutic education program (Annex 1).

#### 2.5.3. Clarke Test

The perception of hypoglycemia was previously measured using the Clarke test [19], which consists of eight questions. A validated version in Spanish will be used for this study. The Spanish version of the Clarke questionnaire has good psychometric properties, and it is a useful instrument for assessing the presence of undetected hypoglycemia in patients with type 1 diabetes mellitus. The Spanish version showed a Cronbach’s coefficient for internal consistency of 0.75, a correlation coefficient for test–retest reliability of 0.81 and correlations of the questionnaire score with the frequency of non-severe and severe hypoglycemia of 0.47 and 0.77, respectively [20]. 

#### 2.5.4. Diabetes Self-Care Activities Scale

Self-care in this study will be measured using the Summary of Diabetes Self-Care Activities (SDSCA). An adapted and validated version in Spanish will be used in this study. This tool has a simple format that is easy for the patient to fill in, which is why it has been widely used in various studies. The Spanish version of the instrument is culturally appropriate, valid and reliable among Spanish patients with diabetes (Cronbach’s alpha = 0.62) [21].

#### 2.5.5. Goldberg Anxiety and Depression Scale

Emotional state will be assessed using the Goldberg Anxiety and Depression Scale. This is a simple and easy test for patients and is composed of two subscales of nine binary (yes/no) items. The whole scale presents a 91% specificity and 86% sensibility. The cut-off point for the anxiety subscale is 4 or more points, and it is 2 or more points for the depression subscale. The Cronbach’s alpha coefficient for the anxiety subscale is 0.75, and for the anxiety subscale it is 0.73. The reliability coefficient for the anxiety subscale is 0.73, and that for the depression subscale is 0.78, both of which are considered acceptable [22]. 

### 2.6. Data Analysis

All data obtained will be analyzed using the statistical tool IBM SPSS 24.0. Baseline data will be compared between groups using chi-square analysis or independent *t*-test. Parametric statistical tests will be used after checking the normal distribution of the sample. Repeated-measures analysis of covariance will be performed, and the interaction effects on each continuous outcome will be calculated. The chi-square test or Fisher’s exact test will be used to determine the differences in glycemic control and self-care between the two groups. Logistic regressions will be performed to determine the influence of the independent variables on the selected dependent variable. The level of statistical significance will be set at a p value of less than or equal to 0.05. 

### 2.7. Ethical Considerations

Upon ethical approval, participants will be recruited during their visits to the endocrinology and nutrition unit of the hospital. The principal investigator will check the eligibility of each potential study candidate. The study will be explained orally to the patient, and a copy of the signed written informed consent document will be offered. All doubts presented by the participant will be resolved, and participants will be able to revoke their consent at any time. Participation will be voluntary, and the data obtained will be completely anonymous for people outside the research team. The researchers on this study obtained the permissions of those responsible for the participating entities and the approval of the corresponding ethics committee.

## 3. Discussion

The proposed study is in line with current recommendations to advocate for and encourage self-management and self-care for patients with chronic diseases in general and diabetes in particular. To the best of our knowledge and according to our bibliographic search, no previous study has analyzed the benefits of a therapeutic education program through glycemic control with a glucose monitoring sensor tool, including aspects of knowledge, mood and self-care.

Diabetes can negatively influence depression, as poor glycemic control can induce negative moods [23]. A nurse-led psychological intervention based on self-management principles reduced depressive symptoms and improved the perception of quality of life in persons with diabetes and co-occurring depression [24]. High levels of anxiety have been linked to poor controls and incorrect diabetes management [25]. Some articles have shown that high levels of distress contribute to poor self-management behaviors and lifestyles and high glycosylated hemoglobin levels. Some modifiable factors such as self-efficacy and social support have contributed to improving levels of distress and depression [26]. Therefore, the researchers for this study decided to measure these parameters in some way before and after the intervention. The Goldberg scale, a tool that measures anxiety and depression through simple questions that are easy for the patient to answer, was chosen.

Psychological states are closely linked with sleep. Fear of hypoglycemia places a major psychological burden on individuals with type 1 diabetes, which can affect sleep quality [27]. After many years of diabetes evolution, the perception of hypoglycemia is lost. In this study, the authors decided to include the Clarke test for hypoglycemia perception. During therapeutic education, it is important to teach the patient about the symptoms of hypoglycemia and how to manage this situation to avoid severe hypoglycemia.

A meta-analysis reported a relationship between HbA1 and sleep quality [28]. However, although glycosylated hemoglobin provides a biomarker for average blood glucose over a 2-3-month period, it does not capture daily blood glucose fluctuations, something that can be observed with the sensor. Previous scientific evidence has shown that individuals can have an optimal HbA1c level (<8%) yet high glucose variability (GV), with glucose levels ranging from 40 to 400 mg in a 24 h period [27]. This is somewhat relevant because it is related to important long-term complications, and inadequate glycemic control is strongly associated with cardiovascular events and mortality [29]. In our hospital, the majority of patients with type 1 diabetes today use the glucose monitoring sensor. Thanks to this tool, the nurse can check glucose levels even at night. There is a parameter called glucose variability that appears on this device, and we will include it in the study data. It will be interesting to see if the education program improves patients’ glucose variability. These results could have important implications for nursing practice. Nurses should assess their patients with type 1 diabetes for knowledge of short- and long-term diabetes complications, diabetes symptoms and self-management practice, as recommended in the American Diabetes Association *Standards of Medical Care* [30]. 

### Limitations

Several questionnaires were selected to analyze the relevance and effectiveness of the program, but the authors made an effort to find validated scales in Spanish, preferably specific to the population with type 1 diabetes. Only one non-validated questionnaire was used to assess the degree of knowledge about the management of diabetes. The researchers found it interesting to include it to identify the training needs of the subjects and the level from which they started. 

One limitation is that possibly that the intervention addresses too many aspects of diabetes diet and self-care in just four sessions. Based on previous experiences in the education classroom of this unit, the sessions are usually well organized, and the patients understand all the knowledge addressed in four days. For this reason, people with cognitive, vision or hearing disorders will be excluded to alleviate this deviation.

For greater data blinding, the personnel who analyze the data will not be the same as those who teach the educational sessions. The nurse educators will be unaware of the study objectives; the questionnaires will be administered by the research nurse. 

## 4. Conclusions

This program is expected to be a tool to increase patients’ knowledge about the management of type 1 diabetes. It is also intended to increase the security and self-confidence of patients, thus improving both their control and their emotional state. In the long term, it will also decrease the risk of serious diabetes complications. 

The advanced practice diabetes nurse plays a key role in self-care and health management education. The patient must be properly trained and independent in the administration of home treatment, in addition to preventing complications derived from poor glycemic control. The results derived from this study can provide a scientific basis for the importance of starting therapeutic education programs with properly organized and updated training. Finally, improving the knowledge of diabetic patients will result in a decrease in health care costs derived from complications, hypoglycemia, ketoacidosis, foot ulcers and errors in insulin administration. It will also benefit the emotional states and quality of life of patients with diabetes.

## Data Availability

Not applicable.
